# Analysis of Enterprise Social Responsibility to Employee Psychological Satisfaction Based on Discriminant Least Square Regression

**DOI:** 10.3389/fpsyg.2022.925010

**Published:** 2022-07-08

**Authors:** Junbao Ren, Ni Zhong

**Affiliations:** ^1^China Construction Bank Shandong Branch, Jinan, China; ^2^Shandong Tudi Development Group Co., Ltd., Financial Management Division, Jinan, China

**Keywords:** enterprise social responsibility, employee psychological satisfaction, discriminant least square regression, feature selection, artificial intelligence

## Abstract

Employee psychological satisfaction is the satisfaction of perception of environmental factors at the psychological and physiological levels, that is, the employees’ subjective response to the work situation. How to enhance employee loyalty and psychological satisfaction has always been a hot issue in theoretical and practical research. With the development of artificial intelligence (AI), many AI methods are widely used to find important factors which have significant influences on the psychological satisfaction of employees. Feature selection methods as one kind of AI models can select discriminant features which have high correlation with the outcome. In this study, we first construct 19 factors from enterprise social responsibility. Then we use a discriminant least square regression model to select most relative factors associating with employee psychological satisfaction. Our experimental results show that the psychological satisfaction of employees is very related to salary, security, welfare, occupational health, and fairness. In addition, we find that discriminant least square regression performs better than the comparison feature selection methods we select, and the selected factors are more in line with our perceptions and expectations.

## Introduction

Enterprise social responsibility is one of the important achievements of human civilization. No matter what kind of social environment and economic system it is in, and no matter what the property rights structure of the enterprise is, the enterprise must take responsibility for the stakeholders and the environment while creating economic benefits (Boyd et al., 2007; Aguirre and Ocampo, 2012; Bauman and Skitka, 2012; Pavlíek and Doucek, 2015). Of course, the connotation of enterprise social responsibility is extremely rich. Different regions with different economic and social development levels and different historical and cultural backgrounds have different understandings of enterprise social responsibility. Therefore, there will be different enterprise social responsibility rules and standards in different countries and regions. Under certain conditions, these rules and standards may also evolve into tools of competition to exclude commercial rivals. In China, state-owned enterprises are an important material and political foundation. As a state-owned enterprise, actively fulfilling social responsibilities and being responsible to stakeholders such as consumers, employees, communities, and the environment are of exemplary significance to the whole society. Over the years, under the active promotion of government departments and extensive attention from all walks of life, state-owned enterprises have the courage to assume their responsibilities and missions, and consciously fulfill their social responsibilities. Unremitting efforts have been made in areas such as legitimate rights and interests, strengthening production safety, and participating in social welfare activities, and have made positive progress. At present, most state-owned enterprises have initiated social responsibility work, released social responsibility reports, and established a responsible social image.

Employee job satisfaction is an important employee attitude, which reflects the degree of employee preference and dislike for their own work (Su and Zhu, 2014; Lin, 2020). Therefore, employee job satisfaction can be regarded as a kind of psychological emotion. In this study, we call it *Employee Psychological Satisfaction*. Because employee psychological satisfaction has an important impact on the production and operation of the enterprise, the competitiveness of the enterprise, and the financial status of the enterprise, managers of various enterprises attach great importance to employee psychological satisfaction. Enterprise managers usually use anonymous questionnaires to obtain the satisfaction level of their employees. By obtaining data on employee psychological satisfaction, enterprises can dynamically observe employee satisfaction levels. If it is found that the level of employee psychological satisfaction has dropped significantly, enterprise managers will use various means to obtain factors that affect the decline of satisfaction, such as deteriorating production conditions, slow wage increases, slow job promotions, and obvious unfair distribution of benefits, etc. Managers find out the most important factors that affect employee psychological satisfaction through surveys, and then take targeted measures to overcome these factors, and conduct evaluation and feedback. In the United States, the human resource management department of most companies will regularly conduct employee psychological satisfaction surveys. For example, the Boeing Company of the United States regularly conducts a questionnaire survey on the psychological satisfaction of its employees in various departments to find and solve problems in a timely manner. Early Boeing employees had very high psychological satisfaction, which led to many beneficial outcomes, such as higher employee innovation levels, and lower turnover intentions, etc. As a result, Boeing’s level of financial performance significantly exceeds that of European Airbus. The former president of Boeing proudly said: “Compared to Airbus, our advantage is simply that our employees have a higher level of psychological satisfaction, however, this advantage is enough to beat any competitor.” In the later stage, Airbus realized the importance of employee psychological satisfaction, and discovered three important factors affecting employee psychological satisfaction in time: the degree of matching between corporate values and employee values, providing employees with promising positions, and employee identity. Airbus takes corresponding measures to effectively improve the psychological satisfaction of employees. This was crucial for Airbus to become a serious competitor to Boeing again later.

With the development of artificial intelligence (AI), many AI methods are widely used to find important factors which have significant influences on the psychological satisfaction of employees. For example, Prentice et al. (2020) explored how emotional and artificial intelligence influences employee retention and performance with a focus on service employees in the hotel industry. The authors found that emotional intelligence has a significant effect on employee retention and performance (Prentice et al., 2020). Bhargava et al. (2021) qualitatively explored working adult’s perceptions of the implementation of robotics, AI, and automation on their job satisfaction, job security, and employability. Feature selection methods as one kind of AI models can select discriminant features which have high correlation with the outcome. According to Miao and Niu (2016); Li et al. (2017), Jiang et al. (2020), and Zhang et al. (2021b), feature selection can be divided into three types: filtering-based type, wrapped-based type and embedded-based type (Nie et al., 2010). Most of the early feature selection algorithms belong to the filtering-based type, which performs feature selection before model training. It means that feature selection and modeling are independent of each other. Wrapped-based feature selection is different from the filtering-based type. Filtering methods do not need to consider modeling, while the wrapped-based type is dependent on modeling. The evaluation criteria for feature subsets are based on the performance of the model to be used. Embedded-based feature selection is to link the feature selection process with the modeling process, and it is a combined type of the filtering-based type and the wrapped-based type. Practice shows that embedded-based feature selection can select the features that are more related to the outcome (Li et al., 2017; Venkatesh and Anuradha, 2019). In this study, we first construct 19 factors from enterprise social responsibility. Then we use a discriminant least square regression model belonging to the embedded-based type to select most relative factors associating with employee psychological satisfaction. Our experimental results show that the psychological satisfaction of employees is very related to salary, security, welfare, occupational health, and fairness. In addition, we find that discriminant least square regression performs better than the comparison feature selection methods we select, and the selected factors are more in line with our perceptions and expectations.

The following sections are organized as follows. In section “Related Work,” we make a survey regarding recent studies. In section “Data and Methods,” we present the method and data used in this study. In section “Data and Methods,” we report the experimental results and in the last section, we discuss and conclude this study.

## Related Work

In the 1970s, with the rapid development and wide attention of corporate social responsibility, more and more scholars began to study the relationship between corporate social responsibility and employee satisfaction, and the relationship between corporate social responsibility and employee loyalty. Since the beginning of the twenty-first century, many scholars in China have paid more and more attention to corporate social responsibility, which has also triggered a research upsurge on the relationship between corporate social responsibility and employee satisfaction.

Foreign scholars have conducted many studies on the relationship between corporate social responsibility and employee satisfaction. On the basis of a deep understanding of exchange theory, scholars such as Eisenberger found that when the organization’s care, support and recognition for employees are experienced by employees themselves, employees will make corresponding behaviors such as positive evaluation and positive recognition of the organization. At the same time, employee satisfaction has improved. In terms of employees’ perceived corporate social responsibility and their own job satisfaction, a survey conducted by the Hudson Institute in 2000 showed that employees who believe that “the company is a responsible company” are 6 times as many employees who believe that “this company is not socially responsible” (Johnston and Packer, 1987). After analyzing a large number of literatures related to organizational identification, Riketta (2005) pointed out that when employees have the emotion of organizational identification, employees will work more actively, and their satisfaction will increase accordingly. After studying the relationship between employee training and employee satisfaction, Chen et al. (2004) found that in today’s fiercely competitive society, employees are more and more eager to receive more professional development training. If enterprises can provide employees with more training opportunities, the employee’s job satisfaction will be greatly improved (Chen et al., 2004). McFarlin and Sweeney (1992) conducted a study on the relationship between the fairness of the compensation system and employee satisfaction and concluded that no matter what the compensation management system is, it will have an impact on employees’ job satisfaction and work attitude.

Wang et al. (2020) conducted research on employees’ perceived corporate social responsibility and their own job satisfaction and concluded that corporate social responsibility to employees, employee satisfaction with job returns, and overall corporate satisfaction have a significant positive impact. Brammer et al. (2007) took three important factors that affecting employee job satisfaction, namely work remuneration, work environment, and work relationship as control variables. Empirical research showed that employees’ perception of leadership’s social responsibility orientation is significantly positively correlated with their satisfaction. Abraham (2001) divided the content of corporate social responsibility into four aspects: enterprise-to-employee, consumer, environment, and charity, and conducted an empirical investigation and concluded that corporate social responsibility is generally positively correlated with employees’ job satisfaction. However, the role of corporate philanthropic responsibility practice is not significant. From the perspective of the impact of corporate social responsibility on organizational behavior, De Stefano and Aloisi (2019) divided the image of corporate social responsibility into three dimensions: labor rights, human rights protection, and management systems, and divided employee job satisfaction into three dimensions: work, employee compensation, and self-development. The organizational citizenship behavior is divided into two dimensions: OCBQ and OCBI. They finally came to a conclusion: the relationship between various dimensions of corporate social responsibility image (labor rights, human rights protection, management system) and employee job satisfaction (job, employee compensation, self-development) and organizational citizenship behavior dimensions (OCBQ, OCBI) have a significant positive correlation; the labor rights and human rights protection dimensions of the corporate social responsibility image have a significant predictive effect on employee job satisfaction and employee organizational citizenship behavior.

From the above literature, we can see that there is a close relationship between corporate social responsibility to employees and employee satisfaction. By summarizing and sorting out relevant literature, there are relatively few studies on the relationship between employee social responsibility and employee satisfaction in state-owned enterprises. The only studies are also some simple linear regression analysis, which cannot explain the research problem in depth, thus affecting the guidance of practical work. Therefore, from the standpoint of enterprise employees, this research will study the specific correlation between multiple variables in corporate social responsibility to employees and employee satisfaction, and calculate the corresponding influencing factors and path coefficients, so as to provide information for state-owned enterprises and even all enterprises. Provide reference to improve the systematic, pertinent and effective performance of employees’ social responsibilities. This will ultimately improve employee job satisfaction, stimulate employee enthusiasm and creativity, and promote the healthy and sustainable development of the enterprise.

## Data and Methods

### Data

The dataset we used in this study was provided by Ying (2018), which was collected from 600 enterprise staffs. In each sample, the enterprise social responsibility was evaluated from 4 dimensions, i.e., economic responsibility, legal liability, moral responsibility, and development commitment responsibility. The employee psychological satisfaction was partitioned into be positive and negative. As for each enterprise social responsibility, it can be further divided into several factors, as shown in Table 1.

**TABLE 1:** Factors of enterprise social responsibility.

Enterprise social responsibility	Factors
Economic responsibility	**X1**: Enterprises formulate reasonable compensation strategy.
	**X2**: Enterprises pay salaries in a timely manner.
	**X3**: Enterprises do not deduct wages without reasons.
	**X4**: Enterprises give reasonable overtime pay to the employees who work overtime.
Legal liability	**X5**: Occupational security rights.
	**X6**: Labor social security rights.
	**X7**: Rest and vacation rights.
	**X8**: Safety and health rights.
	**X9**: Insurance benefit rights.
Moral responsibility	**X10**: Enterprises’ help to vulnerable employee groups.
	**X11**: Enterprises’ concern for retired employees.
	**X12**: Fair and transparent rewards and punishments.
	**X13**: Employee medical examination.
	**X14**: Comfortable working environment for employees.
	**X15**: Enterprises respect employees
	**X16**: Employee mental health assistance.
Development commitment responsibility	**X17**: Staff training and education.
	**X18:** Career planning.
	**X19:** Participation in the management by the employees.

### Methods

#### Discriminant Least Square Regression

In this study, we use discriminant least square regression (DLSR) (Nie et al., 2010) to select some of most relevant factors and analyze these factors to predict employee psychological satisfaction. Suppose factors of enterprise social responsibility can be represented as **X**=[**x**_1,_**x**_2_,,**x**_*n*_] ∈ **R***^d^*×*n*, and employee psychological satisfaction can be expressed as **Y** = [**y**_1,_**y**_2_,,**y**_*n*_]*^T^* ∈ **R**^*n*×*c*^, where *n* represents the size of the training dataset, *c* represents the number of emotion categories, *d* represents the number of factors. The objective function of DLSR is defined as follows,


(1) $$\underset{W}{\text{m}\,i\,n}{||{\text{X}}^{T}\,\text{W}-\text{Y}||}_{2,1}+\lambda \,{||W||}_{2,1\;}$$

where λ is a regularized parameter. Since ||⋅||_2,1_ is a norm, which is a convex function, the objective is also convex. Therefore, the objective function in (1) has and only has a global minimum value. Direct solving (1) seems a little difficult as both terms are non-smooth. Therefore, we firstly change the objective function in (1) into another form, as shown in (2),


(2) $$\underset{W}{\text{m}\,i\,n\,\frac{1}{\lambda }}{||{\text{X}}^{T}\,\text{W}-\text{Y}||}_{2,1}+{||W||}_{2,1}\;$$

The objective function shown in (2) can be further simplified as


(3) $$\underset{W,E}{\text{m}\,i\,n}{||E||}_{2,1}+{||W||}_{2,1}\,s.t.{\text{X}}^{T}\,\text{W}+\lambda \,\text{E}=\text{Y}\;$$

which can be rewritten as the following form shown in (4),


(4) $$\underset{W,E}{\text{m}\,i\,n}{||\left[\begin{array}{c} \text{W}\\ \text{E} \end{array}\right]||}_{2,1}\,s.t.\left[\begin{array}{cc} {\text{X}}^{T} & \lambda \,\text{I} \end{array}\right]\,\left[\begin{array}{c} \text{W}\\ \text{E} \end{array}\right]=\text{Y}\;$$

where **I** is an *n* by *n* identity matrix. Let *m* = *n*+*d*, **A**=[**X***^T^*λ**I**], and **U** = $\left[\begin{array}{c} \text{W}\\ \text{E} \end{array}\right]$, therefore, the objective function shown in (4) can be updated as,


(5) $$\underset{U}{\text{m}\,i\,n}{||U||}_{2,1}\,s.t.\text{A}\,U=\text{Y}\;$$

#### Optimization

To optimize the minimization problem shown in (5), we introduce the Lagrangian function of (5), which can be defined as


(6) $$J\,\left(U\right)={||U||}_{2,1}-\text{T}\,r\,\left(\Lambda^{T}\,\left(\text{A}\,U-\text{Y}\right)\right)\;$$

By setting the derivative of *J*(**U**) with respective to U to zero, that is, $\frac{\partial J\,\left(U\right)}{\partial U}=0$, we have


(7) $$\frac{\partial J\,\left(U\right)}{\partial \text{U}}=2\,\text{D}\,U-{\text{A}}^{T}\,\Lambda =0\;$$

where **D** is a diagonal matrix where each diagonal element can be defined as


(8) $$d_{i\,i}=\frac{1}{2||{\text{u}}^{i}|{|}_{2}\;}\;$$

By multiplying two sides of (7) by **AD**^−**1**^, and considering **A***U* = **Y** in (5), (7) can be simplified into the following form,


(9) $$2\,\text{A}\,U-{\text{AD}}^{-1}\,{\text{A}}^{T}\,\Lambda =0\;$$

(10) $$\Rightarrow 2\,\text{Y}-{\text{AD}}^{-1}\,{\text{A}}^{T}\,\Lambda =0\;$$

(11) $$\Rightarrow \Lambda =2\,{\left({\text{AD}}^{-1}\,{\text{A}}^{T}\right)}^{-1}\,\text{Y}\;$$

Finally, by substituting (11) into (7), we have


(12) $$\text{U}={\text{D}}^{-1}\,{\text{A}}^{T}\,{\left({\text{AD}}^{-1}\,{\text{A}}^{T}\right)}^{-1}\,\text{Y}\;$$

The algorithm steps of DLSR are listed as follows.

**ALGORITHM:** DLSR.

**Input:** **X**=[**x**_1,_**x**_2_,,**x**_*n*_] ∈ **R***^d^*×*n*, **Y** = [**y**_1,_**y**_2_,,**y**_*n*_]*^T^* ∈ **R**^*n*×*c*^ **Output: U**
**Procedure**: Step 1: Use (8) to construct the matrix **D.** Step 2: Construct the matrix **A** by **A** = [**X***^T^*λ**I].** Step 3: Use (11) to construct the matrix **Λ**. Step 4: Use (12) to construct **U.**


## Experimental Studies

### Settings

**FIGURE 1:**
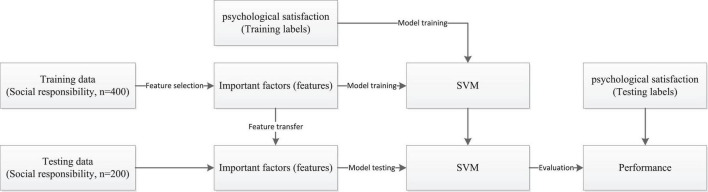
Experimental workflow.

To highlight the feature selection performance of DLSR, five extra methods, i.e., ReliefF (Robnik-Šikonja and Kononenko, 2003; Xia et al., 2020), FscoreRank (fish score rank) (Tsuda et al., 2002; Aksu et al., 2018), *t*-test (Chaves et al., 2009; Wang et al., 2014), information gain (IG) (Roobaert et al., 2006; Azhagusundari and Thanamani, 2013), and mRMR (minimum redundancy maximum relevance) (Mundra and Rajapakse, 2009; Ramírez-Gallego et al., 2017) are introduced for comparison studies. Parameters of these comparison methods are fixed in a default way recommended by the designers. Figure 1 shows the workflow of our experimental studies, where SVM (support vector machine) (Zhang et al., 2021a) with the Gaussian kernel is selected as the final classifier in this study. The performance is evaluated by accuracy.

In the experimental workflow shown in Figure 1, first of all, the dataset regarding enterprise social responsibility is divided into the training data (*n* = 400) and the testing data (*n* = 200). Then DLSR or the comparison models is used to determine the discriminant features based on the training data. The selected discriminant features associating with psychological satisfaction (training labels) are as input to the classifier (SVM) for model training. In the last step, the selected discriminant features are transferred into the testing data and as input to the trained SVM for model testing. After model testing, the testing labels are used for model evaluation.

### Experimental Results

**FIGURE 2:**
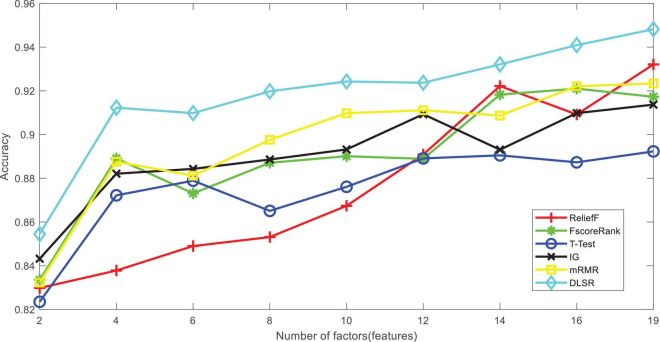
Performance of accuracy against number of enterprise social responsibility factors.

Table 2 and Figure 2 show the performance of enterprise social responsibility to psychological satisfaction in terms of accuracy. The best performance is marked in bold. It can be seen that DLSR wins the best performance, which indicates that the enterprise social responsibility factors (features) determined by DLSR are more relative to the psychological satisfaction of employees.

**TABLE 2:** Psychological satisfaction classification performance of all models in terms of accuracy.

Number of features	ReliefF	FscoreRank	*T*-test	IG	mRMR	DLSR
2	0.8298	0.8335	0.8235	0.8432	0.8323	**0.8545**
4	0.8378	0.8891	0.8722	0.8821	0.8876	**0.9123**
6	0.8490	0.8731	0.8789	0.8843	0.8812	**0.9098**
7	0.8531	0.8871	0.8651	0.8886	0.8976	**0.9198**
10	0.8674	0.8901	0.8761	0.8932	0.9098	**0.9243**
12	0.8909	0.8889	0.8891	0.9093	0.9111	**0.9237**
14	0.9222	0.9183	0.8905	0.8931	0.9087	**0.9321**
16	0.9092	0.9210	0.8873	0.9098	0.9221	**0.9409**
19	0.9321	0.9173	0.8923	0.9137	0.9234	**0.9482**

*The best performance is marked in bold.*

**FIGURE 3:**
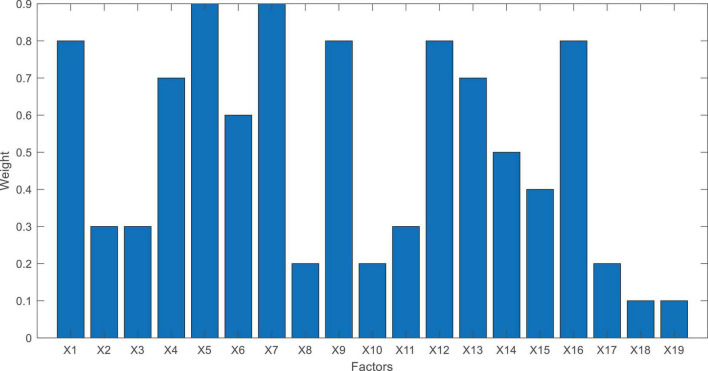
Weight of enterprise social responsibility factor generated by DLSR.

To further determine which factors are more important to the psychological satisfaction of employees, we visualize the transformation matrix **W** in DLSR, as shown in Figure 3. As we stated in section “Related Work” that **W** becomes a sparse matrix after the objective function of DLSR reaches to its global minimum value. Therefore, if values in **W** are bigger, it means that the corresponding factors are more important to the psychological satisfaction of employees. It can be seen from Figure 3 that factors X1, X4, X5, X7, X9, X12, X13, and X16 are more important than others, which means that the psychological satisfaction of employees is very related to salary, security, welfare, occupational health, and fairness.

## Discussion

Job satisfaction refers to the satisfaction of employees’ perception of environmental factors at both psychological and physiological levels, that is, the worker’s subjective response to the work situation. How to enhance employee loyalty and psychological satisfaction has always been a hot issue in theoretical and practical research. At present, domestic and foreign scholars’ research on enterprise social responsibility mainly focuses on the following four aspects. The first is what is enterprise social responsibility; the second is why enterprises should fulfill social responsibility; the third is the impact of fulfilling social responsibility on enterprise financial performance; the fourth is how to promote enterprises to actively fulfill social responsibility. However, most of the existing research starts from the perspective of organizational behavior, and rarely deals with the impact of corporate social responsibility on employee job satisfaction. Friedman (2007) believe that an enterprise is the private property of shareholders, and its main responsibility is to be responsible to shareholders and make profits for shareholders, without having to undertake other social responsibilities. Carroll (1991) gave the definition of enterprise social responsibility: enterprise social responsibility is the expectations that society places on enterprises in economic, legal, ethical, and philanthropic aspects during a specific period. In addition, in order to clarify the object of enterprise social responsibility, Carroll corresponded the economic, legal, ethical, and philanthropic responsibilities of enterprise social responsibility with the corresponding stakeholders, and initially constructed a framework for the relationship between enterprise social responsibility and stakeholders. In recent years, there has been an increasing number of studies advocating and supporting enterprise social responsibility. Through the analysis of 34 empirical literatures on the relationship between enterprise social responsibility and financial performance since 1990, Van Beurden and Gössling (2008) showed that as many as 23 literatures support a significant positive relationship between enterprise social responsibility and financial performance. Lu et al. (2009) further pointed out that enterprise responsibility to the community and the environment positively affects its financial performance, while enterprise responsibility to employees and other stakeholders such as customers can promote the improvement of enterprise non-financial performance. In general, most studies believe that the fulfillment of corporate social responsibility is conducive to establishing corporate image, enhancing enterprise reputation, establishing good relationships with customers and employees, enhancing the attraction to talents, and improving the market competitiveness of enterprises. In this study, we first construct 19 factors from enterprise social responsibility. Then we use a discriminant least square regression model belonging to the embedded-based type to select most relative factors associating with employee psychological satisfaction. Our experimental results show that the psychological satisfaction of employees is very related to salary, security, welfare, occupational health, and fairness. In addition, we find that discriminant least square regression performs better than the comparison feature selection methods we select, and the selected factors are more in line with our perceptions and expectations.

## Conclusion

Employee psychological satisfaction is an important employee attitude, which reflects the degree of employee preference and dislike for their own work. In this study, we first construct 19 factors from enterprise social responsibility. Then we use a discriminant least square regression model belonging to the embedded-based type to select most relative factors associating with employee psychological satisfaction. Our experimental results show that the psychological satisfaction of employees is very related to salary, security, welfare, occupational health, and fairness. In addition, we find that discriminant least square regression performs better than the comparison feature selection methods we select, and the selected factors are more in line with our perceptions and expectations. In our future work, we will develop new model to find the important factors which have significant influences on employee psychological satisfaction.

## Data Availability Statement

The original contributions presented in this study are included in the article/supplementary material, further inquiries can be directed to the corresponding author.

## Ethics Statement

The studies were reviewed and approved by the Ethics Committee of Shandong Tudi Development Group Co., Ltd. The participants provided their written informed consent to participate in this study.

## Author Contributions

NZ contributed on data preprocessing. JR contributed on coding and writing. Both authors contributed to the article and approved the submitted version.

## Conflict of Interest

NZ was employed by the Shandong Tudi Development Group Co., Ltd and JR was employed by the company China Construction Bank Shandong Branch.

## Publisher’s Note

All claims expressed in this article are solely those of the authors and do not necessarily represent those of their affiliated organizations, or those of the publisher, the editors and the reviewers. Any product that may be evaluated in this article, or claim that may be made by its manufacturer, is not guaranteed or endorsed by the publisher.
